# Polysomnographic characteristics of sleep in adults with and without physician-diagnosed atopic dermatitis: results from the Study of Health in Pomerania

**DOI:** 10.1007/s11325-023-02937-7

**Published:** 2023-10-27

**Authors:** Katharina Piontek, Andreas Arnold, Ralf Ewert, Beate Stubbe, Thomas Bremert, Markus Krüger, Ingo Fietze, Henry Völzke, Christian Apfelbacher

**Affiliations:** 1 Institute of Social Medicine and Health Systems Research, Medical Faculty Magdeburg, Leipziger Str. 44, 39120 Magdeburg, Germany; 2 Department of Dermatology, University Medicine Greifswald, Greifswald, Germany; 3 Department of Internal Medicine B – Cardiology, Pneumology, Weaning, Infectious Diseases, Intensive Care Medicine, University Medicine Greifswald, Greifswald, Germany; 4 Department of Oto-Rhino-Laryngology, Phoniatrics and Pedaudiology Division, University Medicine Greifswald, Greifswald, Germany; 5 Department of Prosthetic Dentistry, Gerodontology and Biomaterials, University Medicine Greifswald, Greifswald, Germany; 6 Center of Interdisciplinary Sleep Medicine, Charité-Universitätsmedizin Berlin, Berlin, Germany; 7 Institute for Community Medicine, University Medicine Greifswald, Greifswald, Germany

**Keywords:** Polysomnography, Atopic dermatitis, General-population, Population-based study

## Abstract

**Purpose:**

To analyze sleep characteristics as measured with polysomnography (PSG) in adults from the general population with and without physician-diagnosed atopic dermatitis (AD).

**Methods:**

We analyzed data from participants from the German population-based Study of Health in Pomerania (SHIP) TREND-0. AD was diagnosed in a standardized skin examination. The following polysomnographic parameters were measured: total sleep duration (min), sleep latency (min), wake after sleep onset (WASO; min), rapid eye movement (REM) latency (min), sleep efficiency (%), total number of wakefulness and movement episodes, stages of sleep (%), and apnea-hypopnea index (AHI). Additionally, the subjective sleep quality was assessed using the Pittsburgh Sleep Quality Index (PSQI). We compared sleep characteristics of participants with and without AD.

**Results:**

Among 1187 participants, 47 (4.0%) had AD. We found no differences between participants with and without AD in any of the analyzed PSG parameters except for the total number of wakefulness and movement episodes and the percentage of REM sleep. Participants with AD had a higher number of wakefulness and movement episodes, and a lower proportion of REM sleep compared to those without AD. Regarding subjective sleep parameters, no significant differences were found between participants with and without AD.

**Conclusion:**

Our data do not provide evidence for poor sleep quality in individuals with AD. Major limitations of the study include the unavailability of data on AD severity and the small number of participants with AD. Larger-scaled longitudinal studies considering disease severity and specific AD symptoms with an effect on sleep are required.

## Introduction

Atopic dermatitis (AD) is a chronic inflammatory skin disease characterized by recurrent flexural eczema and pruritus as the leading symptom. AD is related to multiple comorbidities (both atopic and non-atopic) and carries a substantial burden of disease [1, 2]. Sleep disorders are common in AD and are a major factor leading to diminished quality of life [3]. Studies indicate that 33 to 90% of adults with AD experience sleep disturbances. Difficulties falling asleep, frequent nighttime awakenings, early morning awakening, and daytime fatigue are commonly reported problems [3]. Impaired sleep may lead to excessive daytime sleepiness, mood disturbances, and diminished functioning at home and work, thereby increasing the risk for cardiovascular, metabolic, and psychiatric diseases [4]. While previous studies on sleep quality in AD have focused on children, studies among adults are limited [3, 5]. Notably, sleep was evaluated as a secondary outcome using subjective assessments in most studies, and only few investigations have used objective measurements such as polysomnography (PSG), which is considered the gold standard [3]. Data from population-based studies may help to expand knowledge regarding the prevalence and magnitude of sleep disturbances among adults with AD, which is important for health care management. The present study aimed to investigate polysomnographic characteristics in adults from the general population with and without physician-diagnosed AD.

## Materials and methods

### Setting and study population

**Fig. 1:**
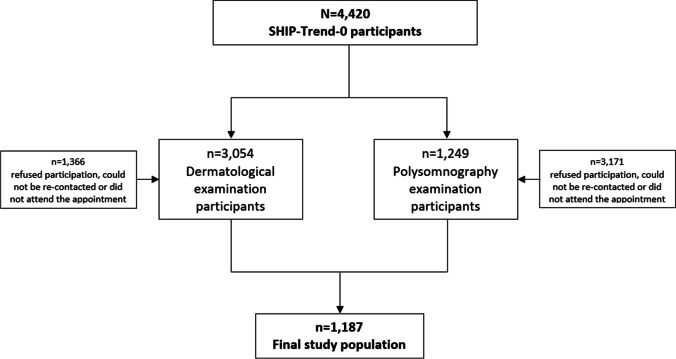
Flow chart of the recruitment procedure

We analyzed data from the population-based cohort Study of Health in Pomerania (SHIP) TREND-0, a baseline examination which was conducted from 2008 to 2012 in West Pomerania, a region in the northeast of Germany. Details on the sampling procedure have been described in detail previously [6]. In brief, a stratified random sample of 10,000 individuals aged 20 to 79 years was drawn from the central population registry in the Federal State of Mecklenburg-Vorpommern. Stratification variables were age, sex, and city/county of residence. The net sample (without migrated or deceased persons) comprised 8826 individuals. Potential participants received a maximum of three written invitations, followed by phone calls and home visits in case of non-response. The final study population included 4420 participants (response 50.1%). SHIP-TREND-0 examinations included a dermatological examination and an overnight PSG, in which 3054 (68%) and 1249 (28%) participated, respectively. For the present analyses, data from 1187 individuals, who had participated both examinations, were included. The recruitment procedure is displayed in Fig. 1.

The data that support the findings of this study are available from the FVCM Transfer Unit for Data and Biomaterials, University Medicine Greifswald, Germany, but restrictions apply to the availability of these data, which were used under license for the current study, and so are not publicly available. Data are however available from the authors upon reasonable request and with permission of the FVCM Transfer Unit for Data and Biomaterials, University Medicine Greifswald, Germany.

### Measures

#### Dermatological examination

AD status was evaluated by experienced, trained physicians in a standardized dermatological examination, which included a personal interview and a skin examination encompassing the whole body, scalp and nails. AD was diagnosed based on the participant’s self-report and the physician’s evaluation.

#### Polysomnography

An overnight PSG was conducted according to the standards of the American Academy of Sleep Medicine (AASM) [7] using ALICE 5 PSG devices (Philips Respironics, Eindhoven, The Netherlands) [8]. The following parameters were analyzed: total sleep duration (minutes), sleep latency (the time from “lights out” that marks the starting of total recording time to the first epoch scored as sleep) (minutes), wake after sleep onset (WASO; periods of wakefulness occurring after defined sleep onset) (minutes), rapid eye movement (REM) latency (the time from sleep onset to the first epoch of REM sleep) (minutes), sleep efficiency (percentage of total time in bed actually spent in sleep), and the total number of wakefulness and movement episodes. Furthermore, the stages of sleep were measured encompassing N1, N2, N3 and REM (percentage). Stages N1 to N3 are considered non-rapid eye movement (NREM) sleep, with a progressively deeper sleep in each stage [9]. REM sleep is associated with dreaming and not considered a restful sleep stage [9]. As an indicator of the severity of sleep apnea, we calculated the apnea-hypopnea index (AHI). The AHI is defined as the number of apneas or hypopneas recorded during the study per hour of sleep. To determine the severity of sleep apnea, the AHI is categorized as follows [10]: < 5 (normal), 5–14 (mild), 15–29 (moderate), and ≥ 30 (severe).

#### Subjective sleep quality

The subjective sleep quality was assessed using the Pittsburgh Sleep Quality Index (PSQI) [11]. The PSQI is a 19-item self-report measure to assess the respondents’ amount of sleep and the extent to which various factors interfere with their sleep in the past month on a four-point Likert-type scale ranging from *not at all* (0) to *three or more times a week* (3). From these 19 items, a global score and the following seven component scores are derived: sleep quality, sleep latency, sleep duration, sleep efficiency, sleep disturbances, sleep medication, and daytime dysfunction due to sleepiness. Each component is scored from 0 to 3, and the total score ranges from 0 to 21, with higher scores indicating poorer sleep quality.

#### Data on demographic and health-related variables

In a standardized, computer-assisted face-to-face interview, we assessed the following data: sex, age, partnership status (having a partner: yes/no), educational level (years of schooling: less than 10 years, 10 years, more than 10 years), net household income in €, current smoking status (dichotomously: yes/no), and average daily consumption (in grams of pure ethanol). Further health-related variables were assessed in a self-administered questionnaire. We measured the individuals’ self-perceived health status using the following question from the Short Form 12 Health Survey (SF-12) [12]: “In general, would you say your health is…” This item was rated on a 5-point Likert scale as either *excellent* (1), *very good* (2), *good* (3), *moderate* (4), or *bad* (5), and analyzed continuously with higher scores indicating a poorer health status. The SF-12 was also used to measure physical and mental health-related quality of life (HRQoL). For this purpose, we considered the four scales measuring physical health (Physical Functioning, Role-Physical, Bodily Pain, General Health), and the four scales measuring mental health (Vitality, Social functioning, Role-Emotional, Mental Health) consisting of six items, respectively. Using a norm-based algorithm ranging from 0 to 100 points, a physical component summary (PCS) score and a mental component summary (MCS) score were calculated with higher scores indicating better physical and mental functioning.

### Statistical analyses

Prior to our data analyses, we performed an analysis of missing data. The AD variable contained no missing data. The proportion of missing values in the PSG variables was 0.5% except for REM latency (2.9%), and the PSQI items had < 1.6% missing values. The demographic variables contained < 0.1% missing data. The percentage of missing values was 0.3% for the variable assessing self-rated general health and < 1.8% for the variables measuring physical and mental HRQoL. Descriptive data analyses were performed. First, we described sociodemographic and health-related characteristics of our study population stratified by AD status. Group differences were analyzed using t-tests for continuous variables and Pearson χ^2^-tests for categorical variables, and data are reported as means (standard deviation) and as frequencies (percentages), respectively. Second, we analyzed differences between individuals with and without AD with respect to sleep parameters obtained from PSG and PSQI by performing linear regression analyses. To account for the potential impact of age and sex  on sleep measures [13], all analyses were adjusted for age and sex. For each variable, the adjusted mean and the corresponding 95% confidence interval (CI) are reported. For effect size estimates, we calculated partial *η*^2^.

Data analyses were conducted using STATA 14.2 (Stata Corporation, College Station, TX, USA). Tests were considered statistically significant at a two-sided *p*-value of < 0.05.

## Results

### Characteristics of the study population

In the total study population of 1187, 47 (4.0%) participants had AD (Table 1). These were less often female, younger, and reported a lower household income compared to those without AD. No significant differences were found between individuals with and without AD concerning health-related variables.

**Table 1:** Characteristics of the study population stratified by the presence of atopic dermatitis

	Total study population	With AD	Without AD	
	*N* = 1187	*n* = 47	*n* = 1140	*p*-value
*Demographic data*				
Female sex, n (% yes)	548 (46.2)	21 (44.7)	527 (46.2)	.044
Age; years (M, SD)	52.8 (14.0)	45.0 (14.5)	53.1 (13.9)	< .001
Having a partner, n (% yes)	933 (78.7)	33 (70.2)	900 (79.0)	.149
School education, n (% yes)				.227
< 10 years	205 (17.3)	7 (14.9)	198 (17.4)	
= 10 years	589 (49.7)	29 (61.7)	560 (49.2)	
> 10 years	392 (33.1)	11 (23.4)	381 (33.5)	
Household income in € (M, SD)	1418.13 (741.68)	1427.39 (744.27)	1197.09 (645.58)	.039
*Health-related data*				
Current smoker, n (% yes)	233 (19.7)	10 (21.3)	223 (19.6)	.774
Alcohol consumption (grams per day), M (SD)	9.0 (12.3)	6.6 (7.3)	9.1 (12.4)	.169
Self-reported general health, M (SD)	2.9 (0.7)	2.9 (0.7)	2.9 (0.7)	.741
Physical HRQoL (SF-12 PCS), M (SD)	47.3 (8.7)	49.4 (7.8)	47.2 (8.7)	.091
Mental HRQoL (SF-12 MCS), M (SD)	52.3 (9.1)	50.0 (9.7)	52.4 (9.1)	.079

Data are reported as means (standard deviation) for continuous variables and as frequencies (percentages) for categorical variables

*AD* atopic dermatitis, *HRQoL* health-related quality of life, *SF-12 PCS* Short Form (12) Health Survey Physical Component Score, *SF-12 MCS* Short Form (12) Health Survey Mental Component Score

### Polysomnography data

Regarding PSG parameters, participants only differed in the total number of wakefulness and movement episodes and REM sleep (Table 2). Participants with AD had a higher number of wakefulness and movement episodes and a lower percentage of REM sleep compared to those without.

**Table 2:** Sleep characteristics obtained from overnight polysomnography

	Total study population (*N* = 1187)	With AD (*n* = 47)	Without AD (*n* = 1140)		
	Means adjusted for age and sex (95% CI)			*p*-value	*η*^2*a*^
Total sleep duration (minutes)	374.0 (370.4; 377.6)	363.4 (345.3; 381.6)	374.4 (370.7; 378.1)	0.245	0.01
Sleep latency (minutes)	15.6 (14.6; 16.6)	17.1 (12.3; 22.0)	15.5 (14.6; 16.5)	0.528	0.01
WASO (minutes)	61.0 (58.6; 63.5)	67.4 (55.0; 79.7)	60.7 (58.3; 63.3)	0.304	0.01
REM latency (minutes)	143.6 (139.1; 148.2)	154.7 (131.3; 178.1)	143.2 (138.6; 147.8)	0.345	0.01
Sleep efficiency (%)	81.4 (80.7; 82.0)	79.7 (76.4; 83.0)	81.5 (80.8; 82.1)	0.304	0.01
Total number of wakefulness and movement episodes	18.4 (17.9; 18.9)	22.1 (19.4; 24.8)	18.2 (17.7; 18.8)	0.007	0.01
Stages of sleep (%)					
N1	27.8 (27.0; 28.6)	30.0 (26.1; 33.8)	27.7 (26.9; 28.5)	0.262	0.01
N2	39.8 (39.1; 40.4)	39.0 (35.8; 42.2)	39.8 (39.1; 40.5)	0.621	0.01
N3	18.7 (18.2; 19.2)	19.1 (16.7; 21.5)	18.7 (18.2; 19.1)	0.715	0.01
REM sleep	13.8 (13.4; 14.1)	12.0 (10.2; 13.7)	13.9 (13.5; 14.2)	0.040	0.01
AHI	10.7 (9.9; 11.5)	8.9 (5.1; 12.7)	10.8 (10.0; 11.6)	0.334	0.01

*AD* atopic dermatitis, *AHI* apnea-hypopnea index, *CI* confidence interval, *REM* rapid eye movement, *WASO* wake after sleep onset

^a^Partial eta squared for comparison of participants with and without AD; 0.01–0.059 small effect, 0.06–0.139 medium effect, ≥ 0.14 large effect

### Data on subjective sleep quality

Analyzing the self-rated sleep quality using PSQI data, no significant differences between participants with and without AD were found (Table 3).

**Table 3:** Subjective sleep quality measured using the Pittsburgh Sleep Quality Index

	Total study population (*N* = 1187)	With AD (*n* = 47)	Without AD (*n* = 1140)		
	Means adjusted for age and sex (95% CI)			*p*-value	*η*^2*a*^
PSQI component (M, SD)					
1. Sleep quality	1.2 (1.2; 1.2)	1.4 (1.2; 1.6)	1.2 (1.2; 1.2)	0.067	0.01
2. Sleep latency	1.2 (1.2; 1.3)	1.4 (1.2; 1.7)	1.2 (1.1; 1.3)	0.115	0.01
3. Sleep duration	1.2 (1.2; 1.3)	1.1 (0.9; 1.4)	1.3 (1.2; 1.3)	0.437	0.01
4. Sleep efficiency	0.9 (0.8; 0.9)	1.1 (0.8; 1.4)	0.9 (0.8; 0.9)	0.197	0.01
5. Sleep disturbances	1.1 (1.1; 1.1)	1.2 (1.1; 1.4)	1.1 (1.1; 1.1)	0.087	0.01
6. Sleep medication	0.2 (0.2; 0.2)	0.3 (0.1; 0.5)	0.2 (0.2; 0.2)	0.145	0.01
7. Daytime dysfunction	0.8 (0.7; 0.8)	0.9 (0.7; 1.1)	0.8 (0.7; 0.8)	0.153	0.01
Global score (M, SD)	6.6 (6.4; 6.8)	7.6 (6.5; 8.6)	6.6 (6.34; 6.8)	0.060	0.01

*AD* atopic dermatitis, *CI* confidence interval, *PSQI* Pittsburgh Sleep Quality Index

^a^Partial eta squared for comparison of participants with and without AD; 0.01–0.059 small effect, 0.06–0.139 medium effect, ≥ 0.14 large effect

## Discussion

This study presents comprehensive data on polysomnographic characteristics of adults from the general population with and without physician-diagnosed AD. All measured parameters were similar between individuals with and without AD except for the number of wakefulness and movement episodes and the percentage of REM sleep.

When comparing sleep parameters as measured in our study with normative values obtained from a meta-analysis of PSG parameters including more than 5000 healthy adults [14], we found lower values in study participants with AD than in the reference population for total sleep duration (363.4 vs. 394.6 min) and sleep efficiency (79.7 vs. 85.7%), but a higher value for sleep latency (17.1 vs. 15.4 min). They also showed higher scores for WASO (67.4 vs. 48.2 min), indicating a higher extent of sleep fragmentation. Regarding the stages of sleep, participants with AD showed higher scores for N1 (30.0 vs. 7.9%), but lower scores for N2 (38.9 vs. 51.4%) and REM sleep (12.0 vs. 19.0%) compared to the reference population. Scores for N3 stage were comparable (19.1 vs. 20.4%). In participants with AD, but also in the total study population, higher AHI scores in comparison with the reference population were found (8.9 vs. 2.9 and 10.7 vs. 2.9, respectively).

Few previous studies investigating sleep characteristics in individuals with AD have applied objective measures such as PSG or actigraphy. Actigraphy is a non-invasive method for the assessment of sleep-wake cycles by involving a small wrist-worn device, and measures have been found to be highly correlated with PSG findings in individuals with AD [3]. With respect to the interpretation of the parameters measured in the present study, comparative data are extremely limited. A recently published meta-analysis based on case-control and cohort studies using PSG or actigraphy in adults and children with AD identified seven studies with 173 patients with AD and 112 controls [13]. Overall analyses showed that patients with AD had decreased total sleep time and sleep efficiency, and prolonged WASO and REM latency compared to controls. Among the studies included in this review, two had performed actigraphy in adults with moderate to severe AD. In contrast to our findings, the first study including 14 patients and 14 controls found that patients with AD slept less, awoke more often, and spent more time awake during these waking episodes, resulting in lower sleep efficiency compared to controls [15]. Similarly, the second study based on data from 15 patients and 19 controls showed that patients with AD had lower total sleep time and lower sleep efficiency, and higher WASO compared to controls [16].

Our analyses revealed that participants with AD had a higher number of wakefulness and movement episodes than participants without AD. Likewise, a study investigating nocturnal movements using accelerometers among adults with pruritic dermatoses including AD found that patients had twice as many movements as controls [17]. REM sleep, the “dreaming” state, most often occurs in the early morning hours of a normal sleep cycle and is considered essential for memory consolidation [18]. As an indicator of sleep impairment, we found that participants with AD had a lower percentage of REM sleep compared to those without.

The association of AD with obstructive sleep apnea (OSA) is not well investigated yet. A recent cross-sectional investigation among 2648 U.S. adults reported an association of OSA symptoms with self-reported AD [19]. According to our descriptive data, mean AHI scores among participants with and without AD correspond to mild OSA. This finding probably reflects a peculiarity of our study population since individuals with sleep problems were potentially more likely to participate in the PSG examination.

There is strong evidence to suggest that disease severity and specific AD symptoms such as pruritus and scratching are significant contributors to sleep disturbances in adult patients with AD [4]. For example, a study applying PSG and actigraphy in 20 adults with AD revealed that increasing disease severity was related to more scratching and reduced sleep quality [20]. Another study among 35 adult patients with AD used PSG and an infrared video camera system to investigate nocturnal scratching over 112 nights [21]. That study found longer durations of scratching and being awake after a scratching in patients than in controls. Moreover, numerous studies based on self-reports demonstrated an association of disease severity and scratching with sleep disturbances in adults with AD [22–27].

In view of aspects of feasibility, self-reported measures are important tools for the assessment of the quality and patterns of sleep, and the PSQI is a well-established and widely used instrument for this purpose. In contrast to previous studies applying the PSQI in patients with AD [15, 23], no significant differences regarding subjective sleep parameters between individuals with and without AD were found in the present study population. Notably, patients with AD are supposed to have higher disease severity than individuals with AD from the general population, which is associated to poor sleep quality [13, 27]. Therefore, the comparability of data from patient populations with data from the general population in this regard is limited.

When interpreting the existing evidence, several significant conceptual and methodological concerns should be discussed. Limiting the validity of the results and the comparability of the findings, most studies were designed as cross-sectional investigations with small sample sizes, and show strong heterogeneity regarding the included study populations, the methods used for evaluation of AD status and disease severity, and the availability of data on AD symptoms with impact on sleep quality. Notably, the majority of studies have analyzed sleep as secondary outcome, and self-reports, which might be subject to recall bias, were commonly applied. However, although PSG provides objective data, it is time- and cost-consuming, not widely available, and can be uncomfortable and burdensome for the patient [5], which limits its application in large trials. Besides these methodological issues, the pathophysiology of sleep disturbances in AD is extremely complex and difficult to map and measure in studies. In this regard, several contributing factors have been discussed including disease flares with itch and scratching, sleep habits such as co-sleeping and behavioral insomnia, environmental factors, melatonin dysregulation, nocturnal pruritus due to the circadian rhythm of the skin, and cytokine dysregulation [3].

Taken together, the results of the present study do not provide evidence for sleep disturbances as measured with PSG in adults with AD from the general population. As an indicator of sleep difficulties, we found a higher number of movement and wakefulness episodes and a lower proportion of REM sleep among individuals with AD compared to those without. Along with the existing literature, our study provides important directions for future research. To further elucidate the relationship between disease severity, AD symptoms, and sleep characteristics, larger-scaled longitudinal studies allowing for the assessment of the temporal course of disease severity and of symptoms with impact on sleep such as nocturnal pruritus and scratching are indispensable. Concerning the assessment of sleep characteristics, the application of actigraphy in combination with self-reported data on both sleep quality and symptom intensity might be indicated in view of feasibility and costs.

### Strengths and limitations

The present study is based on data from a population-based study applying high quality standards of the performed examinations including standard operating procedures, certification procedures, and quality reports, which enhances the reliability of the results. AD status of study participants was evaluated by physicians in a standardized skin examination, which is considered the gold standard in epidemiological surveys [28]. PSG provides objective data on sleep characteristics, and the PSQI is a well-established and widely used questionnaire to measure the subjective quality and patterns of sleep in adults [29]. Also, important limitations should be noted. First, our study is based on data from a single PSG recording, and the so-called “first night effect” (FNE) might have impacted our results. It has been argued that first night data may reflect a period of adaptation that is unrepresentative of usual sleep patterns [30], and previous studies showed that the FNE is characterized by decreased sleep duration and decreased sleep quality [31, 32]. Therefore, we cannot rule out that the FNE might have biased our results. Second, data on AD severity and symptoms such as nocturnal pruritus and scratching, which are considered critical determinants of sleep quality in individuals with AD, were not available. Third, since AD is characterized by often seasonal fluctuations and temporal remission for a certain time in a year, one-time skin examination as applied in the present study might exclude mild or transient AD [28], leading to potential underestimation of AD cases. Fourth, the overall sample size was small. Only 26.9% of the SHIP-TREND-0 participants took part in both the dermatological examination and the PSG, which must be evaluated in the context of the broad examination program in SHIP lasting several hours. Also, the potential time delay between the dermatological examination and the PSG measure might have impacted our findings since participation in both investigations was not in close temporal connection in every case.

## Conclusion

We did not find evidence for poor sleep quality as evaluated using PSG parameters in individuals with AD from the general population. Future longitudinal studies applying objective measures of sleep such as actigraphy in combination with self-reported data on sleep characteristics and AD symptoms are indicated to further investigate the relationship of AD with sleep quality.
